# Fatigue Behavior of 3D Braided Composites Containing an Open-Hole

**DOI:** 10.3390/polym12092147

**Published:** 2020-09-21

**Authors:** Shuangqiang Liang, Qihong Zhou, Haiyang Mei, Ge Chen, Frank Ko

**Affiliations:** 1 Department of Mechanical Engineering, Donghua University, Shanghai 200051, China; lsq66122662@163.com (S.L.); zhouqihong@dhu.edu.cn (Q.Z.); 2 Department of Material Engineering, University of British Columbia, Vancouver, BC V6T 1Z4, Canada; frank.ko@dhu.edu.cn; 3 School of Mechatronic Engineering, Harbin Institute of Technology, Harbin 150001, China; meihaiyang2018@gmail.com

**Keywords:** textiles, 3D braided, notched, fatigue, failure

## Abstract

The static and dynamic mechanical performances of notched and un-notched 3D braided composites were studied. The effect of longitudinal laid-in yarn was investigated in comparison with low braiding angle composites. The specimens were fatigue tested for up to millions of cycles, and the residual strength of the samples that survived millions of cycles was tested. The cross-section of the 3D braided specimens was observed after fatigue loading. It was found that the static and fatigue properties of low angle 3D braided behaved better than longitudinally reinforced 3D braided composites. For failure behavior, pure braids contain damage better and show less damage area than the braids with longitudinal yarns under fatigue loading. More cracks occurred in the 3D braided specimen with axial yarn cross-section along the longitudinal and transverse direction.

## 1. Introduction

Three-dimensional braided composites, due to their proven damage tolerance, delamination free properties, and the ability to generate complex network structures in composites, continue to attract academic and industrial researchers’ interest in a wide variety of matrix types, covering carbon (CCC), ceramics (CMC), metal (MMC) and polymer (PMC) matrix composites [1].

Gause and Alper [2] evaluated the static and dynamic mechanical properties of 3D braided composites, covering the open hole static strength and tensile fatigue behavior. They demonstrated for the first time that 3D braided composites are insensitive to notches (openings). Macander [3] demonstrated that 3D braided composites have greater damage tolerance than laminates. Ko [4,5] first proposed a unit cell for four-step braided composites. The Fabric Geometry Model (FGM) was presented for predicting strength and modulus of 3D braided composite. Chen [6] found that the excellent pull-out strength gave 3D braided composite the potential to develop composite nuts and space joint nodes which connect several rods through thread in various directions. Li [7] found that for 3D braided composite, all the structural parameters can be expressed as functions of the preform of pitch length for certain constituent yarns and braiding plans, which is controlled by the process operating conditions.

In recent years, research has been published on the fatigue behavior of braided composite. Portanova [8,9] examined the fatigue resistance of un-notched 3D braided composites after an impact, the residual fatigue life and post-impact strength of 3D braided composite was sufficient to satisfy composite damage tolerance design criteria. It is found that a design using [30°/0°] braid as an integral part of composite wings, such as spars or frames, will have sufficient fatigue strength. Carvelli [10] studied the tensile fatigue behavior of 3D rotary carbon/epoxy composites, and interesting results from their study exhibit that 3D braided composites are the only textile composites with better properties than Unidirectional-Carbon fiber reinforced polymers (UD-CFRP). 

In the last decade, a few researchers used the numerical method for analyzing the static and dynamic characteristics of 3D braided composites, avoiding time-consuming and high-cost experiments [11,12,13,14,15]. Yu et al. [16] developed composites unit cell characterization tool, a secondary development on the finite element commercial platform. Xu et al. [17,18] proposed a meso-scale progressive fatigue damage model in Finite Element Analysis (FEA) for textile-reinforced composites, and separate failure criteria of yarn and matrix were established. Zeng et al. [19,20] developed a new multiscale approach for studying thermomechanical properties of 3D braided composites. Zhang [21,22] presented a meso-scale progressive damage model for 3D braided composite under fatigue tensile load, and their method mainly applies to unit cell, as fatigue failure criteria and material performance degradation were proposed for yarn and matrix separately. 

For open-hole fatigue study on composites, Stinchcomb evaluated the damage accumulation around circular holes and sharp notches in angle-ply laminates subjected to tension cycling load, and the accumulation of fatigue damage was studied by determining the residual strength [23,24]. Daggumati et al. [25,26] studied the fatigue behavior of notched and uncut 3D woven composites. In comparison to the orthogonal structure, the angle interlocking structure exhibited higher open hole static strength and a smaller damage area in the open hole tensile fatigue test. Numerous studies have been undertaken on fatigue properties of open-hole laminates. Muc and Nixon [27,28] conducted the study on fatigue damage growth monitoring for notched laminated composite, and the non-destructive testing methods were applied in their studies, such as X-ray and digital image correlation. Sudarsono et al. [29] studied the effect of openings on the fatigue properties of quasi isotropic initial CFRPs manufactured in autoclaves and by pressing, and compared them with standard continuous CFRP. They reported that the fatigue strength of molded CFRP was about 20% of that of autoclave, slightly higher than that of continuous carbon fiber cloth. Recently, the progressive damage analysis method was implemented to study the notched fatigue behavior of notched composite laminates, and the commercial tools were also developed, such as Helius PFA, AlphaSTAR, and Multiscale Design Systems [30].

The fatigue performance of undamaged 3D composites is weaker than that of undamaged UD CFRP. However, z-yarns do slow down the crack propagation caused by edge delamination, also improving the fatigue performance of 3D composites relative to UD composites when the samples contain macro defects such as holes [31,32]. From the literature review, none included open-hole fatigue behavior of 3D braided composites. Considering that 3D braided composites possess excellent open-hole strength retention, the authors also stressed the need for information on the fatigue behavior of open-hole 3D braided composites. Accordingly, this work is aimed at studying the opening effect of two kinds of 3D braided composites under static and fatigue loads. 

## 2. Experimental 

### 2.1. Material Manufacturing

**Figure 1:**
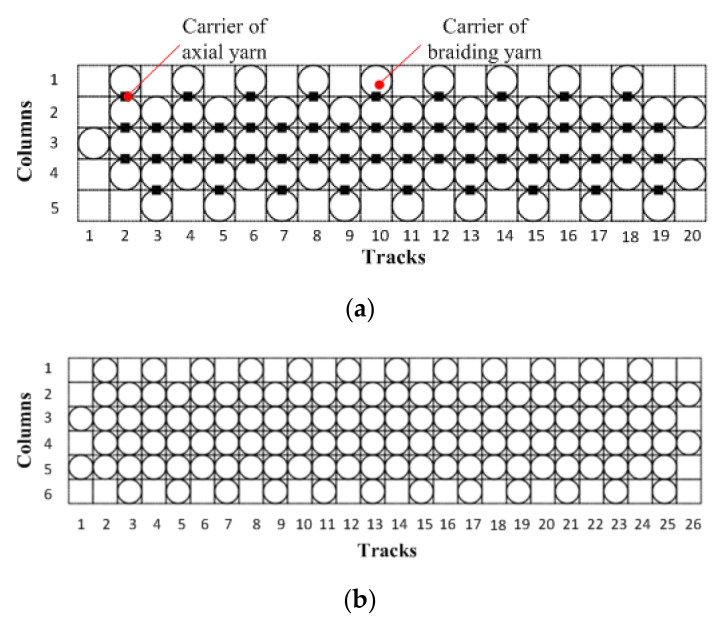
Loom set up with two types of preforms: (**a**) Style I; (**b**) Style II.

Three-dimensional braided preforms were produced using AS4 6K carbon yarns on a Cartesian four-step three-dimensional knitting machine. Two kinds of braided composites were prepared and tested. Style I was the basic (1 × 1) pattern. Style II incorporated 42% longitudinal lay-in yarns. The surface braiding angle of 3D braided fabric was ±12°. To reach the desired width of the standard test coupon and thus avoid cutting the edges of the cured composite panel, 129 (including 54 fixed carriers for 0° axial yarn, 4 columns by 18 tracks) and 124 fiber carriers (6 columns by 26 tracks) were used to separately manufacture 3D braided preforms for Style I and Style II coupons, respectively. Figure 1 shows the loom set up in a rectangular configuration for two types of preforms.

**Figure 2:**
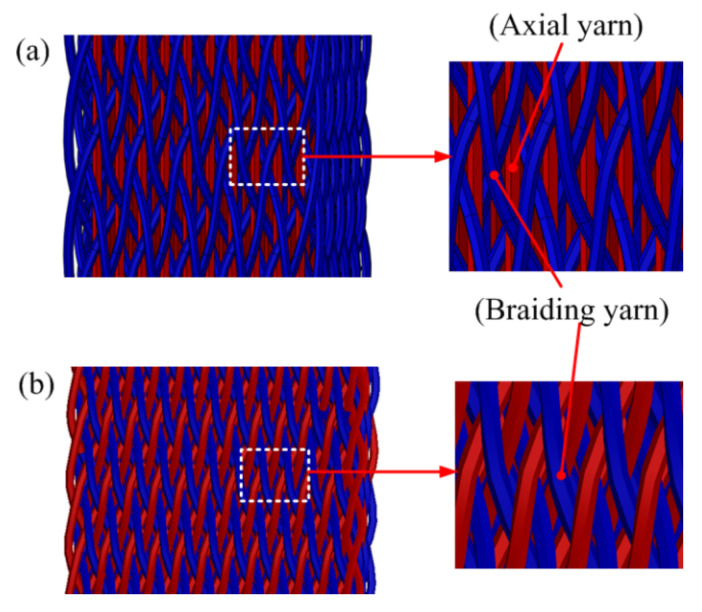
Schematic of the yarn architecture for two types of 3D braided preforms: (**a**) Style I; (**b**) Style II.

The produced 3D braided preforms and the corresponding schematic of fiber architecture are shown in Figure 2. Figure 2a,b show Style I and Style II braided preform, separately.

The preform was then thoroughly wetted by an epoxy resin system consisting of West System 105 and 209 hardeners (mix ratio = 3.68:1 by weight) and molded using a closed mold for at least 24 h and cured under constant pressure at room temperature. The completely cured composite with a rectangular cross-section was released from the mold. The properties of the resin and carbon fiber used in this study are shown in Table 1.

**Figure 3:**
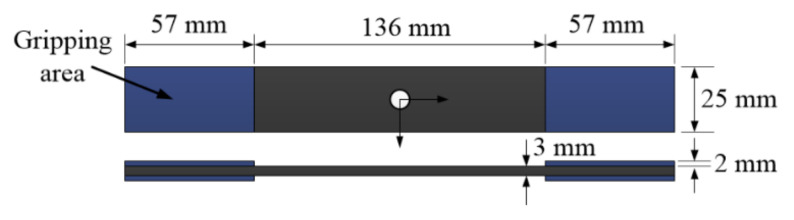
Geometry and dimensions of un-notched specimen.

The geometry and dimensions of the composite specimen with holes are exhibited in Figure 3, with the target width and thickness for all specimens (notched and un-notched) being 25 mm and 3 mm, respectively. A special CBN diamond coring bit was used to drill the open hole in the center of the sample. The width diameter ratio of the naked eye size is 5.

According to the previous work [33], the pore content is also determined by the weighing method. The voids measured for type I and type II specimens were 3% and 4%, respectively. The fiber volume fraction of the type I sample is 59% and that of the type II sample is 62%. 

### 2.2. Quasi-Static and Fatigue Test Procedures

The static un-notched and notched tensile strength were determined according to the ASTM D3039 and ASTM D5766, respectively. For static tests, 5 samples were tested on an Instron tensile tester to obtain the mean tensile strength, with a crosshead speed of 2 mm/min. The un-notched and notched compressive strength was also examined, followed by ASTM D3410 and ASTM D6484 separately, at a rate of 1.5 mm/min. 

For the tension-tension fatigue study, the specimens were tested according to the ASTM D3479 standard, constant amplitude sinusoidal cyclic tension-tension load was applied at R-ratios of 0.1, and at least 10 samples were tested to obtain the S–N curve for each group. All tests were performed on an MTS closed-loop servo-hydraulic testing machine equipped with a self-aligning hydraulic fixture, employing a clamping pressure of about 4 MPa for transferring the load to the specimen by friction. A cycling rate of 3 Hz was chosen to minimize the effects of specimen heating. Each test performed 10^6^ cycles or failed completely. Specimens surviving 10^6^ cycles were residual strength tested. All tests were carried out at room temperature. 

During testing, the surface of the un-notched fatigue specimens was monitored visually to determine the initiation and progression of damage. A Digital Image Correlation (DIC) system was used to obtain the full in-plane filed on the surface of the 3D braided composite of each quasi-static test, as well as the post-fatigue strength test. 

## 3. Results and Discussion

### 3.1. Yarn Cross Section 

**Figure 4:**
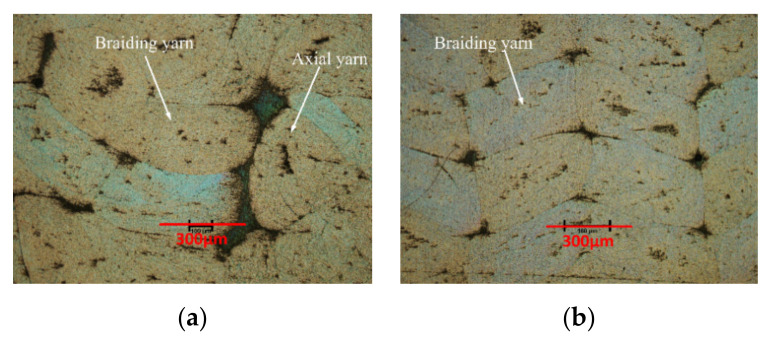
Cross-sectional area photos of 3D braided composites: (**a**) Style I; (**b**) Style II.

**Figure 5:**
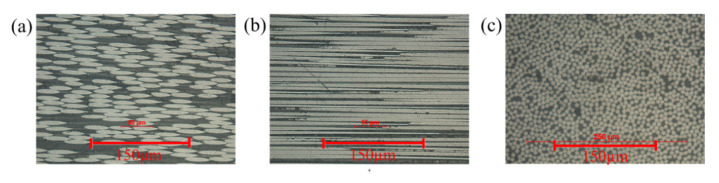
Photographs of fibre cross-section of 3-D braided composite: (**a**) the longitudinal cross section of braided yarn; (**b**) the longitudinal cross-section of laid-in yarn; (**c**) the transverse cross section of axial yarn.

The transverse cross-sections (perpendicular to load direction) of the two types of specimens are exhibited in Figure 4. The yarn cross-section of pure woven fabrics is usually more uniform than that of the axial yarns of type I. Photomicrographs of sections of the braided specimen yarn at fiber scale are shown in Figure 5. The longitudinal cross section (parallel to the load direction) of braiding yarn of Style I is exhibited in Figure 5a. The longitudinal cross section of laid in yarn of Style I is shown in Figure 5b, and the 0 tows tend to be uniformly straight and distributed throughout the thickness. The transverse fiber cross section of all braiding yarn is shown in Figure 5c. 

### 3.2. Open–Hole Quasi-Static Results

**Figure 6:**
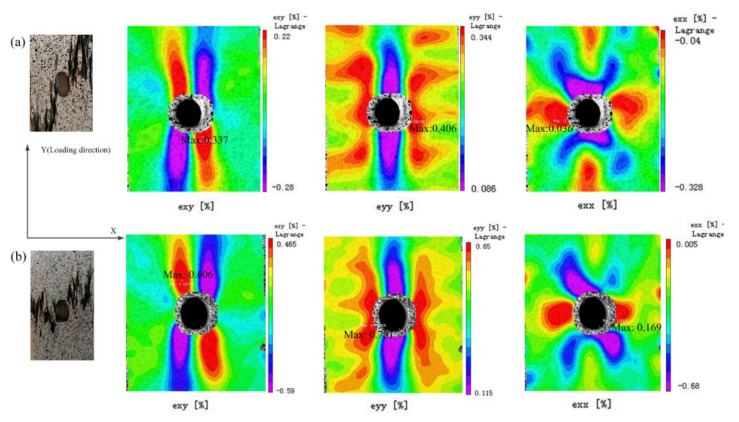
Digital image correlation (DIC) strain field of two 3D braided specimens under tensile loading: (**a**) Style I, (**b**) Style II.

With textile composites, the strain on the surface will vary within the unit cell of the fiber architecture, where unit cell refers to the smallest repeating geometry of the textile architecture [8]. The DIC technique was used in conjunction with all quasi-static tests to obtain the full in-plane strain field on the outer face of the 3D braided composite. The strain fields $\varepsilon_{yx}$, $\varepsilon_{yy}\;$(loading direction), and $\varepsilon_{xx}\;$(transverse direction) of two types of 3D braided composites containing an open hole under the same loading are shown in Figure 6. The images on the left show the final catastrophic failure of two types of specimens. Both braids behave almost identically; the strain distribution is usually concentrated near the hole boundary, whereas the strain value is barely different under the same loading.

Table 2 summarizes the tensile, compressive, Open hole tension (OHT), and Open hole compression (OHC) strengths of 3D braided composites and compares them with published outcomes for 3D braided composites and laminated composites. Comparing Style I and Style II structure, no matter under tension and/or compression loading, notched and/or un-notched, the value deviation is not significant. The braids with 42% axial yarn did not show higher strength in comparison to the basic pattern, implying that the structure and properties of the fabric were not improved by adding the axial yarn at the weaving angle of ±12°.

In comparison to layered composites, the OHT strength retention rate of 3D braided composites is higher. For example, with the uncut 18 ply (0±20°) laminate [34], after drilling, the tensile strength is similar to the existing results and can only maintain 58% of the tensile strength. The un-notched tensile strength of 0° Hexcel laminate [35] is 2205 MPa, and the strength decreases sharply to 438 MPa after drilling, which is only half of that of the open hole 3D braided composite. The results show that the tensile strength of the two kinds of 3D braided composites is 74–80%, so the ultimate notched tensile strength of 3D braided composites is much higher than that of laminated composites.

Generally, 3D braided composites can also maintain high OHC strength, except in the case of 24 layers [2], and their original compressive strength is very low. Even the non-notched compressive strength is lower than that of laminates, and the notched compressive strength is similar to that of notched laminates. According to the failure mode of the three-dimensional braided composite under a compression load, there is no buckling phenomenon, which is mainly due to the overall structure of the three-dimensional braided composite, which is conducive to improving the damage resistance of the material. On the other hand, for laminated plates, natural delamination in compressive buckling can lead to premature failure of the structure. 

In comparison with laminates, our results confirm Gause’s finding [2] that the notch sensitivity of 3D braided composites is lower than that of laminated composites. Thus, considering the advantages of 3D braids, it will be an attractive candidate for structural application.

### 3.3. Open Hole Tension–Tension Fatigue Results 

**Figure 7:**
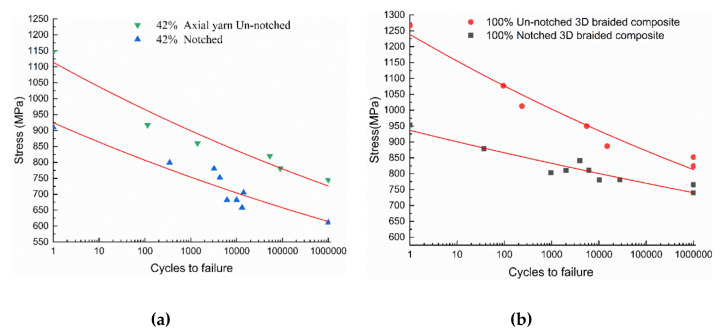
Tension-tension fatigue results: (**a**) life to failure; (**b**) S–N curve, R = 0.1.

The fatigue life curves of notched and un-notched 3D braided composites under tension-tension cyclic load are shown in Figure 7. Each data point represents a sample. A semi-logarithmic function was adopted here to fit the data:

(1) $$\sigma =aLogN_{f}+b$$

where σ represents the maximum applied stress, and $N_{f}^{}$ represents number of failure cycles. The value of parameters a and b is summarized in Table 3.

Fatigue tests of 3D braided composites involve different maximum stress levels in the cycle. From the fatigue life results of 3D braided composites in Figure 7, the fatigue limit of the two kinds of un-notched braided composites is about 65% of the static strength. For notched specimens, fatigue limits happened at about 65% for Style I and 78% for Style II of the respective static strengths. Using the same ratio, pure braided composites tend to show better fatigue life compared to the specimen with longitudinal axial yarn. 

**Figure 8:**
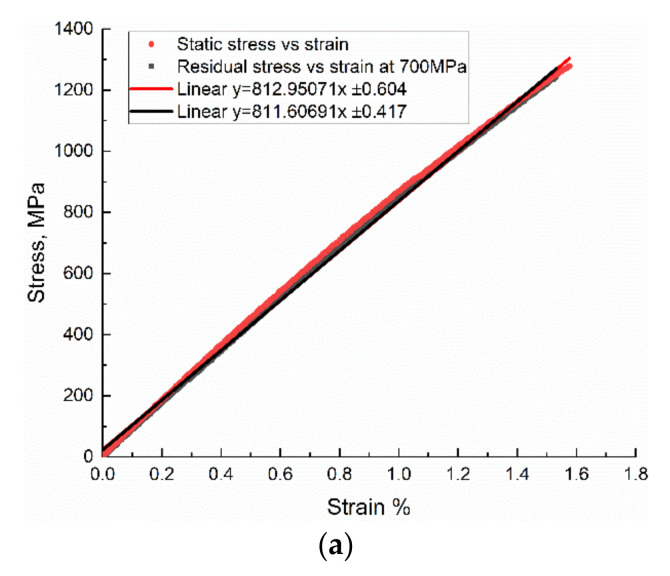

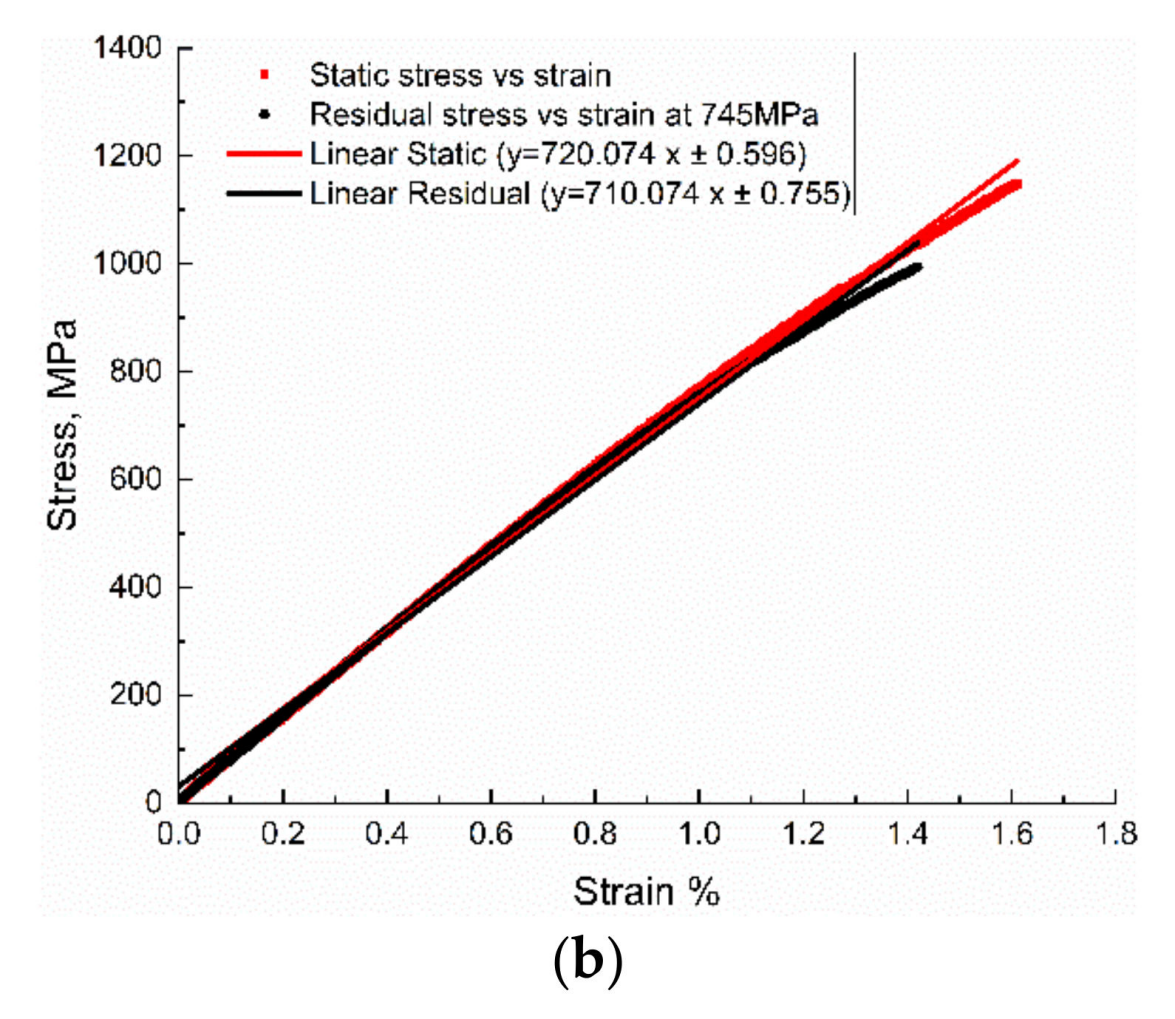
Comparison of stress–strain curves between the original composite specimen and the fatigue test specimen: (**a**) Style I; (**b**) Style II.

For measuring the residual strength of specimens after fatigue, quasi-static tests were performed on the specimen surviving 106 cycles at 700 MPa stress level at 3 Hz. Figure 8 compares the stress–strain behavior of the original sample and the coupon after fatigue loading. The residual strength test exhibits that fatigue loading does not show much influence on the coupon’s modulus, nor the strength.

### 3.4. Failure Mechanism

The failure behavior of 3D braided composites under static load can be obtained from previous studies. The two kinds of notchless 3D braided specimens show different failure behaviors. Under the tensile load, the pure braided fabric is prone to cracking, while the sample with 42% axial yarn is not. For the notched specimen, the crack propagates along the braiding angle from the hole edge to a certain distance, and then propagates between the transverse yarns, resulting in the failure of the specimen. However, obvious cracks still appear in the pure braided specimen, but not in the axial yarn sample. 

Under cyclic loading, some edge surface damage can be observed in the middle of service life, but it seems that there is no significant increase in width or thickness. The matrix crack is formed in the resin-rich area, and the braided tow near the hole extends along the length of the tow on the surface of the sample. The material shows brittle behavior. The fracture of two specimens is very sudden, and there are no visible or audible cracks before failure. In addition, the stress span between infinite fatigue life and failure is very narrow, about 50 MPa in 1000 cycles. 

**Figure 9:**
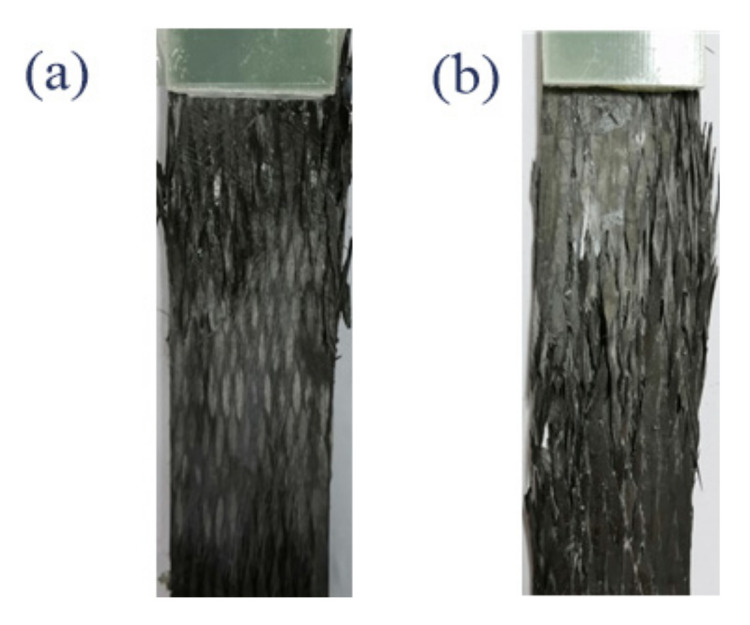
Un-notched specimen photos after fatigue test: (**a**) Style I; (**b**) Style II.

Figure 9 shows the occurring macroscopic fatigue failure of the two types of un-notched 3D braided composite specimens, majorly concentrated in the end pull ring region. The failure behavior of un-notched specimens under fatigue and static loading is different. Unlike notched samples, the damage is usually initiated near the notch, whereas the damage of the un-notched specimen occurred randomly between the end tabs. It should be noted that due to the stress concentration existing underneath the tabs along the longitudinal direction, failure tended to occur near the tabs or inside the tabs during the fatigue test, and the results were not counted. 

**Figure 10:**
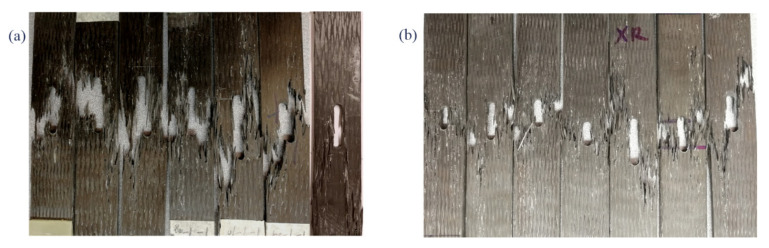
Macro-scale fatigue failure of the notched specimen after fatigue test: (**a**) Style I; (**b**) Style II.

The failure of the notched 3D braided composite under tension-tension fatigue load is shown in Figure 10. Both types show similar failure mechanisms. Early in life, cracks showed up on the surface of the specimens around the hole and along the braiding yarn which, mainly because of the resin rich area, broke first, but did not grow across the coupons. Thereafter, there was almost no change during the entirety of the cycles until the specimens catastrophically broke. There were two main types of failure behavior macroscopically, one was fiber breaking transversely along the hole, and the other one was a crack that traversed the hole along the braiding angle. 

**Figure 11:**
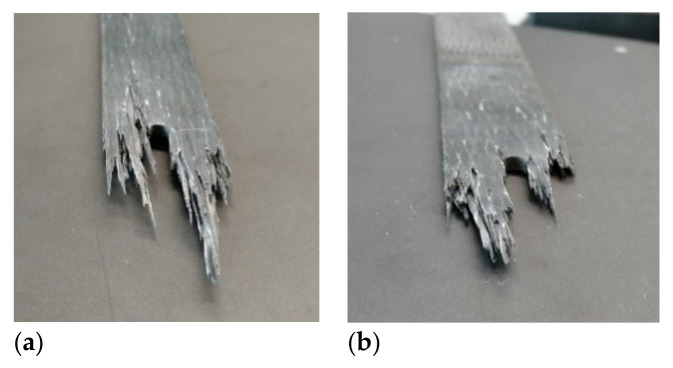
Fracture morphology of notched specimen after fatigue test: (**a**) Style I; (**b**) Style II.

Under tension-tension fatigue load, the notched 3D braided composites exhibited a ‘toothbrush’ fracture surface, which was different from the static case. For Style I, the crack tended to follow the longitudinal yarns and the specimen usually failed with a large damaged area; however, the Style II structure could stop crack propagation significantly and contained less damaged area. Under fatigue loading, damages accumulated in the coupon, inducing matrix crack and decrease in fiber strength, finally causing the specimen to break suddenly at the threshold value. Comparing two types of braided composites, the Style II showed transverse breaks with a small damaged area, whereas Style I showed an explosively failure surface. The fracture surface of the notched specimen after fatigue test is shown in Figure 11.

The notchless and notched fatigue failure mechanisms of 3D braided composites are totally different with laminates. For laminates, damage propagation from notches and holes in T-T fatigue occurs almost exclusively along the vertical (stress) direction [23,24].

**Figure 12:**
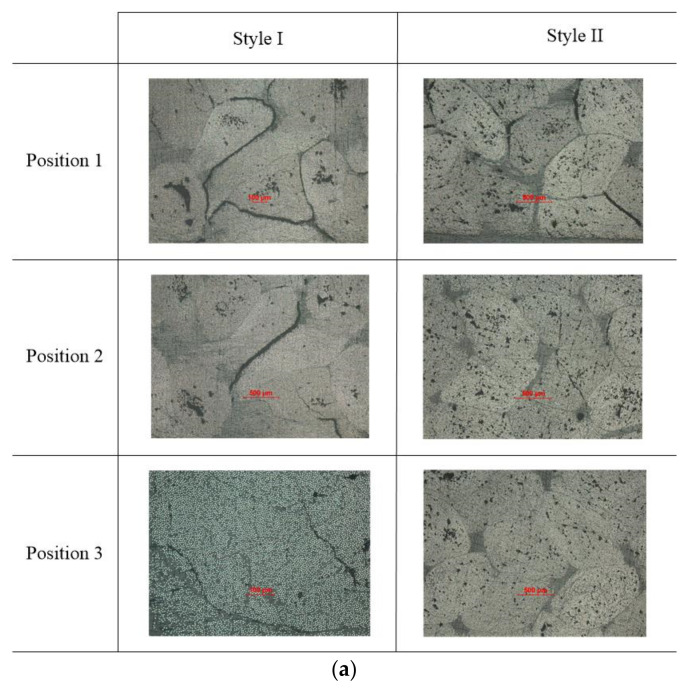

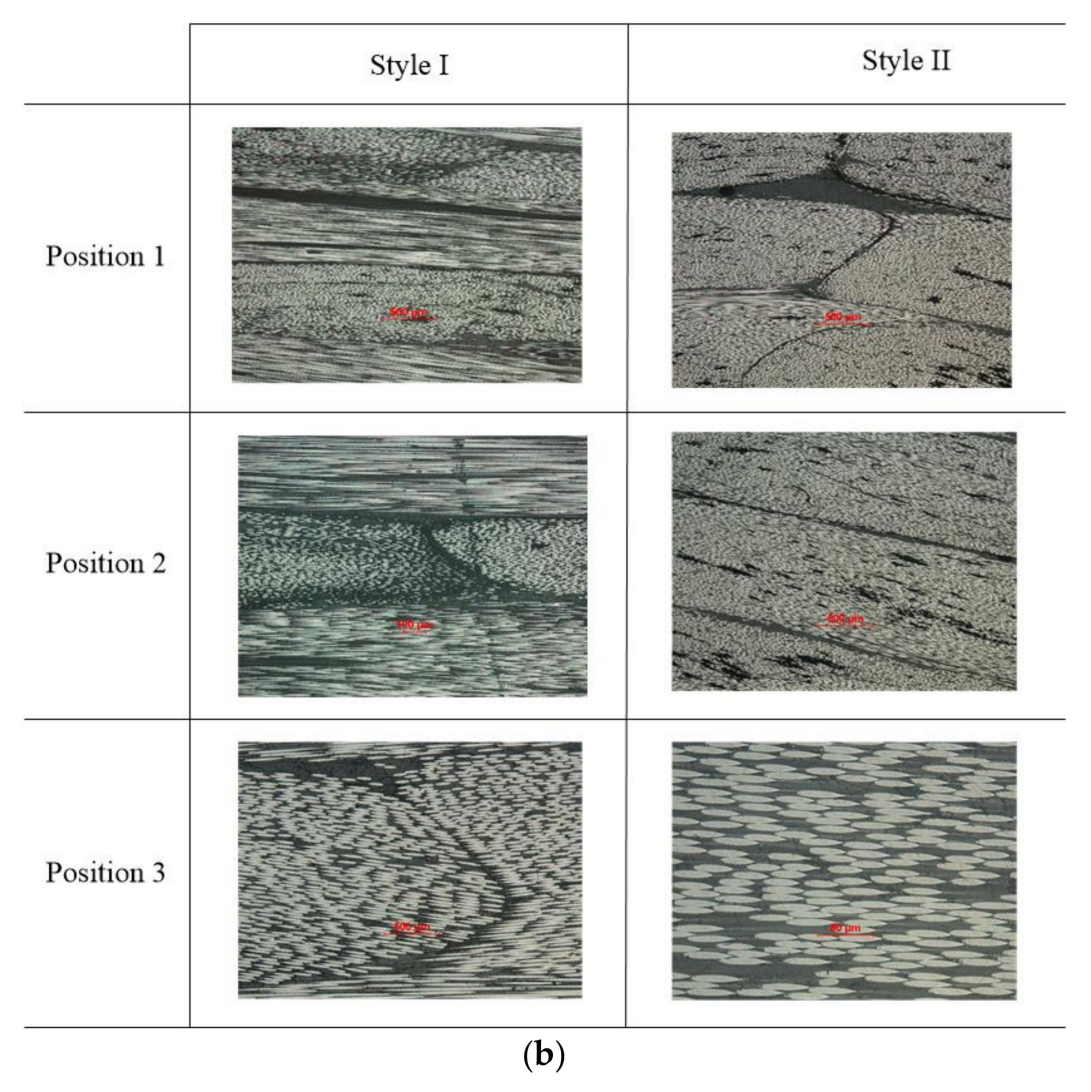
The cross-sectional area photos of notched 3D braided composite: (**a**) transverse section; (**b**) longitudinal section.

Cross-sections of two notched braided coupons after the same fatigue loading are shown in Figure 12. There are increasing cracks when the cut position gets close to the hole (from Position 3 to Position 1). From the transverse section (perpendicular to load direction), the crack begins at the resin enrichment zone and develops along the matrix yarn interface, as shown in Figure 12a. From the longitudinal section (parallel to the load direction), cracks tend to travel along the longitudinal yarn in Style I sample, and the braiding yarn can stop the crack propagating along the loading direction, as shown in Figure 12b. Comparing two types of sample, more cracks show up in the Style I open-hole specimen than Style II, which means pure braided composite contains damage better.

**Figure 13:**
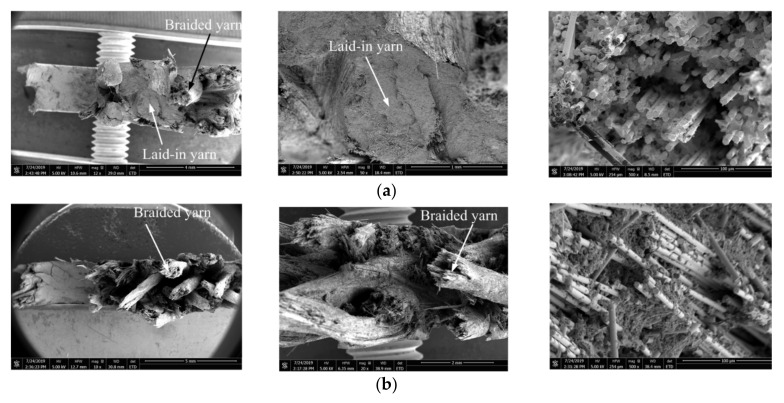
The fracture surface of the notched specimen after fatigue test: (**a**) Style I; (**b**) Style II.

The SEM image of the notch specimen fracture is exhibited in Figure 13. Two types of specimens were examined after fatigue, and it was discovered that the fibers were pulled out and fractured with serious matrix damage. The difference between the two styles of braided coupons is laid-in yarns show regular fracture surface, as shown in Figure 13a, whereas this is not true for braided yarns.

## 4. Conclusions

The fatigue properties of three-dimensional braided composites with and without holes were studied. To the authors’ knowledge, this study is the only one to evaluate the hole effects on the fatigue performances of 3D braided composites. The static properties of the two types of 3D braided composites behaved similarly. The static results confirm that the notch sensitivity of 3D braided composites is lower than that of laminated composites. The fatigue results show that pure notched braided composites have better fatigue resistance than braided composite with axial yarn. Under the tension-tension fatigue load, the notched 3D braided composites exhibited a ’toothbrush’ fracture surface, which was different from the static case. The two types of braided composites behaved similarly in failure mechanisms under tension-tension fatigue loading. Style I tended to fail following the longitudinal yarns with large failure area and an explosive failure surface, while Style II usually transversely broke with small damaged area and contained damage better. More cracks occurred in the Style I specimen, both in the longitudinal and transverse direction. Cracks usually developed along the resin rich area and matrix–yarn interfaces. 

## Figures and Tables

**Table 1:** Properties of matrix and carbon fiber.

Properties	Matrix	AS4-6k
Tensile modulus, GPa	2.74	231
Tensile strength, MPa	51	4447
Strain, %	3.6%	1.7
Density, g/m^3^	1.16	1.78

**Table 2:** The strength of three-dimensional braided laminated composites.

		Tension			Compression			
		*V_f_*	Without Hole/MPa	With Hole/MPa	Strength Retention%	Without Hole/MPa	With Hole/MPa	Strength Retention%
**3-D Braided**	Style I (0° ± 12°)	59	1173 ± 61	910 ± 57	80	589 ± 53	368 ± 61	62
	Style II (± 12°)	62	1279 ± 80	951 ± 45	74	596 ± 56	406 ± 33	68
	C12K/3501 (0° ± 20°) [2]	60	668	661	99	429	314	73
	PEEK/AS4 (0° ± 20°) [34]	60	586	463	79	-	259	-
**Laminates**	Hexcel Ply 8552 [35]	60	2205	432	20	-	-	-
	18-ply APC-2 [(0 ± 20°)_4_, 0]_s_ [34]	60	1081	628	58	646	363	56
	24-ply AS43501 [−45/0/+ 45/90]_s_ [2]	60	911	445	49	420	403	96
	16-ply (0° ± 20°) [36]	63	703	472	67	665	398	60

**Table 3:** The value of fitting parameters.

	a	b
100% un-notched	1113	−0.031
100% notched	930.27	−0.0312
42% axial un-notched	1238.7	−0.031
42% axial notched	936.6	−0.017
